# Assessing the role of organic food supply chain traceability on food safety and consumer wellbeing: A mediated-moderation investigation

**DOI:** 10.3389/fpsyg.2022.1073376

**Published:** 2022-12-01

**Authors:** Zhou Guanqi, Mudassir Husnain

**Affiliations:** ^1^School of Economics, Yunnan University, Kunming, China; ^2^Division of Management and Administrative Sciences, UE Business School, University of Education, Lahore, Pakistan

**Keywords:** organic food supply chain traceability, food safety trust, consumer wellbeing, consumer awareness of organic food, organic food, dynamic capabilities theory

## Abstract

**Introduction/Purpose:**

Drawing on dynamic capabilities theory, this study offers a comprehensive framework for examining the relationships between organic food supply chain traceability, consumer wellbeing, and food safety trust based in organic food sector. The study also explores how consumer awareness of organic food influences the relationship between consumer wellbeing and food safety. We also look at a mediated moderation mechanism in the relationships stated earlier.

**Methods:**

Using a survey as the primary data collecting method, a total of 221 usable responses were collected. To test the hypothesized relationships between all variables, SPSS PROCESS Macro 3.3 is utilized.

**Results:**

The findings show that the organic food traceability OFSC affect food safety trust and food safety and consumer wellbeing are positively associated, while consumer awareness of organic food acts as a moderator on the relationship between food safety trust and consumer wellbeing. Also consumer awareness conditionally influences the strength of the indirect relationship between OFST and consumer wellbeing via food safety, thereby indicates a mediated moderation mechanism among the study variables.

**Discussion/Implications:**

This study clarifies how consumer food safety trust and organic food traceability can enhance their wellbeing. It contributes to the theory of dynamic capabilities as well as organic traceable product marketing strategies.

## Introduction

China a sizable agricultural nation with ideal climate conditions and a wealth of species resources has significant production advantages for organic products. Based on data from China’s National Bureau of Statistics (Sun et al., 2019), 995.03 million tons of organic and agricultural goods were produced in 2019, making up 54.48% of all agricultural output (1826.55 million tons). Green, healthful, and nutrient-dense are the features of organic products (Francois et al., 2020), which people like very much. However, due to the limited storage period and low storage temperature requirements for a number of organic products, food safety accidents are quite likely to happen (Hu et al., 2013).

Organic and veggie agricultural products have experienced a rise in domestic and international safety incidents recently. Such as the China “poisonous ginger” incident (Li et al., 2020), *Listeria* contamination of “Hami melon” in the United States (Desai et al., 2019), and the *Escherichia coli* outbreak in Germany (Luber, 2014), which have seriously damaged the majority of people’s health. As a result, the state places a high value on the ability to track the food supply chain, and nations have strengthened traceability management by passing pertinent laws and regulations. The 2002 General Food Law that the European Union adopted Coppens et al. (2006) specifies that in order to recall products promptly and accurately and to provide information to customers, a thorough traceability system must be implemented in the food business. China’s Food Safety Law, which was put into effect in 2009 (Jintao, 2012), which states that in order to assure food traceability, food producers and operators should set up a food safety traceability system. All food and food-related businesses now face the problem of being “traceable,” and the traceability system has developed into a useful tool for quality control in the supply chain for organic products (Aung and Chang, 2014; Yang et al., 2014; Zhang et al., 2019). When purchasing organic food, consumers have high standards for its quality and depend on certification agencies to both confirm this quality and offer details about the items’ sourcing. However, there are a number of drawbacks with organic food traceability, including issues with organic labeling, certification fraud, and worries about the transparency of food information. There is currently demand on the global food supply chain to give information on environmental effect, food fraud, quality, and safety (Underwood et al., 2013). Consumer demand for high-quality food with safety concerns has risen as a result of several food crises that have occurred in recent years (Schleenbecker and Hamm, 2013). Furthermore, the globalization of the food ecosystem has resulted in an increase in the distances that food travels from producer to consumer (“Farm to Fork”; Lehtinen, 2017). Consumers are paying particular attention to the transparency of the food supply chain for a variety of reasons, including dietary concerns over food ingredients as well as social and environmental issues involved in the production of products (Mangla et al., 2021; Rasool et al., 2021). Previous research has suggested that a lack of government regulation, knowledge asymmetry among stakeholders, and manufacturing management incompetence are all variables that contribute to food safety mishaps (Kendall et al., 2019). Therefore, it is crucial to give consumers more useful information about food in order to regain their trust and faith (Zhang et al., 2020). In the organic food supply chain, knowing a food origin is extremely crucial because it might reveal the usage of pesticides, genetically modified organisms, safety risks, and environmental or carbon impact.

By default, consumers are an essential link in any supply chain (SC). Various supply chain definitions state (Chopra and Meindl, 2007) that most importantly, the entire supply chain should serve the consumer, who is the end-user of the good or service produced and delivered *via* the supply chain. This is in accordance with the supply chain management idea. Consumers today place a high priority on food safety, wanting high-quality food with clear information and assurances of its safety (Aung and Chang, 2014). From the standpoint of the consumer, food traceability systems can lessen information asymmetry, establish safety governance across regional boundaries, and assist in fostering greater confidence in food safety to provide greater consumer wellbeing and satisfaction (Wang et al., 2021). Consumer confidence in the food chain is boosted through traceability and safety, which also contribute to trust-building, assurance, and peace of mind. A traceability system’s implementation and benefits depend on how concerned customers are about food safety; therefore there is a growing need for systems that give clear details about the quality and security of food supply chains. When food traceability is applied across the whole supply chain and involves all relevant parties, it has the potential to yield the highest benefits (Zhu, 2017). As a result, there is a conceptual connection between the traceability of organic food, food safety, and consumer wellbeing. We consider general traceability procedures to be crucial aspects of an organization’s resource management (Garcia-Torres et al., 2021), and their favorable effect on consumers is probably a result of the improvement of food safety regulations. Thus, the association between traceability methods and consumer wellbeing may be mediated by improvements in organic food safety standards.

It is insufficient to simply link traceability practices to favorable results. Examining potential moderators in the link between these constructs has been recommended by researchers (Chang et al., 2016; Irfan et al., 2019). Organic food safety and awareness toward organic food are strongly related. The term “awareness” toward organic foods refers to the understanding and exploration of the traits and qualities that set them apart from their conventional equivalents (freshness, safety, certification, labeling, nutritious, etc.; Ghali, 2019; Le-Anh and Nguyen-To, 2020). The impact of food safety role on consumer wellbeing may also be affected by level of awareness toward organic food (Nguyen et al., 2019). In other words, if the company wants to expand the positive effects of its traceability processes, it could need consumer awareness that can strengthen these relationships. Therefore, when discussing the relationship among traceability practices, food safety concerns, and consumer wellbeing, this paper intends to further analyze the moderating effect of awareness toward organic food on the aforementioned constructs, which is important for understanding the boundaries of the traceability practices. Prior work primarily focused on how to build traceability systems or consumers’ preferences for traceability with specific product supply chains, but rarely addressed the results of applying traceability techniques (Hastig and Sodhi, 2020; Tan et al., 2021; Qian et al., 2022). Furthermore, marketers should give consumers a good reason to choose or buy their products (Berger, 2019), for instance, by increasing perceived value (Iqbal et al., 2021). To better understand consumer expectations regarding organic food supply chain traceability practices, it is therefore essential to understand the safety trust of organic food traceability. Therefore such understanding helps producers and marketers of traceable organic foods develop more informed marketing strategies (Cavite et al., 2021). Going beyond economic impacts to address customer happiness and social value of traceability, this research seeks to investigate how supply chain firms employ traceability technologies to increase consumer consumption values, often results consumer wellbeing.

We address a number of research gaps in this study. In the first place, we add to the scant literature on the direct role of traceability in enhancing customer wellbeing. Second, the role of trust in food safety as a mediator in this relationship is still largely unknown; this study is the first empirical one to look at the potential indirect impact of trust in food safety on the relationship between traceability procedures and consumer wellbeing. Finally, this study looks more closely at the contextual factors that contribute to the link between traceability practices and consumer wellbeing and confirms that high consumer awareness of organic food with food safety trust, has a stronger impact on consumer wellbeing.

## Literature review and hypothesis development

The present study’s theoretical framework draws on theory of dynamic capabilities. The theory of dynamic capabilities states that companies with high dynamic capabilities are better able to recognize emerging market possibilities and combine knowledge resources across many technical domains, ultimately triggering organizational performance improvement activities (O’Reilly III and Tushman, 2008; Beske et al., 2014). It has been suggested that employing sustainability practices enables businesses to keep a handle on and track the food supply chain, as well as implement capabilities to gain a competitive edge and satisfy customer demands. Thus, we consider traceability procedures to be crucial forms of resource management for enterprises (Engelseth et al., 2014), and firm’s potential performance, i.e., customer satisfaction and wellbeing, are most likely due to the enhancement of the organization’s capabilities, i.e., in the form of enhanced organic food safety standards.

### Relationship between organic food supply chain traceability, food safety, and consumer wellbeing

Traceability is the ability to trace and track the history, application, or location of that which is being considered through all stages, including production, processing, and distribution. Traceability was first proposed as a goal of quality management and quality assurance in the ISO 8402 1994 standard (Olsen and Borit, 2013; Centobelli et al., 2018). Traceability is now crucial in order to strengthen the gaps in firm procedures (Dandage et al., 2017), a distinctive asset that gives a company a competitive edge (Epelbaum and Martinez, 2014), and knowledge sources with a focus on value (Engelseth et al., 2014) for a firm. Additionally, several studies show that processes like vertical integration and process reengineering benefit from traceability (Thompson et al., 2005). As a result, we characterize the FSC’s traceability practice as a component of input (supplier), process (FC), and output (consumer) traceability. The process of gathering and tracking data between the FC and its major suppliers is known as input traceability. Process traceability refers to the centralized administration and coordination functions of the Traceability System as well as the traceability procedures associated with FC processes. The activity of gathering and tracking data, including customer needs and market trends frequently related to the quality and origins of food products, is known as output traceability.

Implementing such OFSC procedures enables businesses to adapt to changing consumer preferences, lifestyles, and brand loyalty for organic food products (Matzembacher et al., 2018) and additionally reduces supply chain costs and risk, assuring safe and healthy consumption (Angulo and Gil, 2007). A customer-driven approach is a claim made by every business. However, the multiple value-chain actors, both internal and external, such as suppliers, merchants, and R&D managers, perceive their customers’ needs differently, which causes loss of focus. Therefore, there is a need for frameworks and more research that may assist organic food businesses in creating customer happiness and wellbeing-focused marketing plans (Simons, 2014). Another type of customer-driven supply chain method is product modularity and consumer-driven customization. Earlier research indicated that businesses may effectively tell consumers about safe food by influencing their awareness of the system’s traceability and attitudes, which in turn affects their willingness to pay (Dionysis et al., 2022). According to research on the linkages between sustainability, operations, and marketing, increased customer satisfaction may result from food traceability and configurations that increase demand. They also examine a link between modularity and environmentally friendly products, which could improve sustainable consumption (Ülkü and Hsuan, 2017). Based on these arguments, we developed the following hypothesis:

*H1:* Organic food supply chain (OFSC) is positively related to consumer wellbeing.

Food safety is the level of assurance that a food product will not result in illness or harm while being made, served, or consumed (Matzembacher et al., 2018). Information asymmetry is one of the issues that affect food safety from an economic perspective. Of course, in the absence of good traceability, suppliers frequently engage in opportunistic behavior to take advantage of information asymmetries with customers (Srivastava and Dashora, 2022). FSC traceability, investors, and local administration entities must work closely together to reduce safety hazards. In a broader sense, achieving food safety requires commitment from management and decision-makers on matters of food safety, collaboration and partnership between local governments, shareholders, and nations, allocation of enough capital, and accountability from all interested parties (Uddin et al., 2020). According to Liu et al. (2019) and Garaus and Treiblmaier (2021) consumer perceptions of food safety uncertainty are lowered by product diagnosticity (i.e., the extent to which traceability systems enable consumers to precisely evaluate product attributes, including safety), as well as consumer trust in food supply chain traceability. In this study, we infer that in organic food supply chain traceability system, product factors primarily include traceable food safety, emotional factors primarily include consumers’ perceptions of traceable food reliability, and cognitive factors primarily include consumers’ perceptions of information quality and product diagnosticity. OFSC traceability has emerged as a key tool for producers to show consumers the quality of their organic products (Yin et al., 2016). Based on these arguments we developed the following hypothesis:

*H2:* Organic food supply chain (OFSC) traceability is positively related to organic food safety.

The problem of food safety, quality, and environmental protection, as well as the market’s globalization, have all contributed to a dramatic increase in consumer demand for food traceability during the past 10 years (Yu and Qiao, 2017). Food safety problems seriously endanger customer confidence, human health, food firm earnings, the growth of food industry, and even the reputation of the country abroad (Yu and Qiao, 2017). It should not be surprised that several earlier studies revealed that customers saw buying organic food as a risky concern given consumer concerns about the reliability of organic food (Ashraf et al., 2018; Hsu et al., 2018). As a result, consumer perceptions about the trustworthiness of organic food should be expected to influence their purchasing decisions. Transparency in the food industry promotes consumer confidence, ensures food safety, and encourages them to purchase reliable goods (Zhao et al., 2015; Galvez et al., 2018). Because of investment in their brand identities, food chains and marketers naturally want to safeguard them. They should therefore design and enforce the controls that guarantee their products do, in fact, live up to consumer expectations for quality and safety. Also, organic food consumption has a lower environmental impact, thus prominently contributing to consumer psychological wellbeing (Manning et al., 2019). Consumers want openness and effective accountability in the procedures for ensuring the safety of their food in order to understand the sources and methods used from the farm to fork and ultimately boost their faith in wellbeing. Therefore we propose that:

*H3:* Food safety is positively related to consumer wellbeing.

### Mediating role of food safety

Organizational knowledge resources and the process of organizational learning and sharing are the foundations for changes and advancements of food safety procedures. The major components of traceability procedures include enhancing traceability resources and encouraging the sharing of product information. Epelbaum and Martinez (2014) and Feil et al. (2020) showed that once traceability techniques are transformed into advantages in resources and capabilities, i.e., enhanced safety standards, such capabilities can fuel performance increase. Liu et al. (2013) point out that “customers are not consuming items, but value,” demonstrating that consumers’ judgment of their value prior to purchasing products is a key prerequisite for the occurrence of this consumer behavior. Because of this, organic food chains must develop their distinctive capabilities that go above and beyond their safety criteria in order to achieve an overall performance outcome, for example in terms of consumer wellbeing (Esteki et al., 2019). The more social capital businesses possess and the more resources they can access, the more long-lasting advantages they can create and the more successfully they will operate (Wilden and Gudergan, 2015; Cappa et al., 2021). Therefore, this study suggests that the impact of traceability practices on consumer wellbeing is likely to be mediated through the food safety capabilities. Therefore, we propose the following hypotheses:

*H4:* Food safety mediates the relationship between food supply chain traceability and consumer wellbeing.

### Moderating role of awareness toward organic food

The term “awareness” toward organic foods refers to the understanding and exploration of the traits and qualities that set them apart from their conventional counterparts (freshness, safety, certification, labeling, nutritious, etc.; Ghali, 2019; Song and Morgan, 2019; Le-Anh and Nguyen-To, 2020). In the context of supply chain traceability, consumer awareness of organic food products can strengthen the positive impact of food safety on consumer wellbeing. Based on dynamic capabilities theory, Firms with strict organic food safety standards are better able to adapt to changes in customer product demands and the direction of technological progress in an environment that is highly volatile (Charan and Panghal, 2018; Matzembacher et al., 2018; Zhou et al., 2022). Contrarily, in a technologically stable environment, customer demands and technological advancements are more consistent, consumers have fewer demands regarding product safety, and organizations do not require highly dynamic capabilities to raise consumer awareness of organic food (Makkonen et al., 2014). The degree of consumer awareness of organic foods may also have an impact on how food safety affects consumer wellbeing. In other words, the deeper the consumer’s awareness of the benefits of organic food products, the greater level of consumer wellbeing anticipated due to enhanced food safety standards. Therefore we proposed that:

*H5:* Awareness toward organic food moderates the relationship between the food safety and consumer wellbeing such that the relationship is stronger when consumers have high level of awareness toward organic food products.

**Figure 1:**
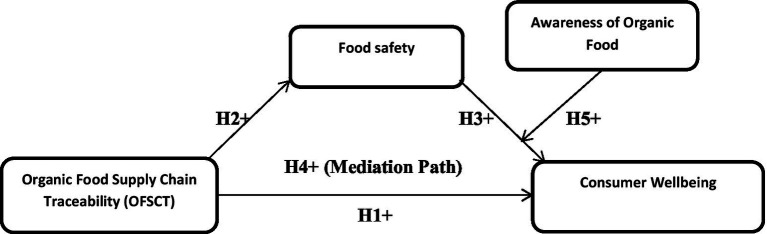
Theoretical framework.

Since we hypothesize that consumer awareness moderates the relationship between food safety and consumer wellbeing, the consumer awareness might conditionally influence the strength of the indirect relationship between OFST and consumer wellbeing *via* food safety, thereby indicating a mediated-moderation configuration between the study’s variables. As depicted in Figure 1, organic food supply chain traceability as an independent variable has been proposed to have a positive effect on consumer wellbeing through food safety trust, while awareness of organic food moderates the positive relationship of food safety trust and consumer wellbeing.

*H6:* Consumer awareness moderates the association among OFST and consumer wellbeing via food safety in such a way that the association will be higher in the case of high consumer awareness.

## Materials and methods

A standardized questionnaire that was distributed as a survey was used for the investigation. Data were gathered in many locations and at different stages in order to prevent favoring any particular set of respondents. The research team asked professionals and academics with relevant backgrounds to change the questionnaire’s wording. The study team conducted the investigation in collaboration with the local Chinese Bureaus of Commerce. Using purposive sampling The Bureaus of Commerce had a database of Xian, Shenzhen, Chengdu, and Shandong organic food firms. To increase the sample’s representativeness, we chose businesses from various areas. Regarding ownership, sample companies include state-owned enterprises, private enterprises, and enterprises with foreign investment. There were 95 companies chosen in all, and these four regions were chosen because of their extensive expertise with FSC traceability techniques and applications.

Two phases of the surveys were completed. In the initial study, 450 questionnaires were given out to assess traceability procedures and pertinent organizational demographics. Middle and senior managers responded to the traceability practices questionnaire that was given to the management in these companies. Four hundred and thirty-nine survey responses were received. Consumers who often buy organic food from these chosen firms were emailed surveys evaluating food safety trust, consumer awareness of organic food, and consumer wellbeing. In this phase, 259 responses were received, and after removing incomplete responses, 221 usable surveys were found, yielding a response rate of 49%.

### Measures

OFST was measured using 15-item scale developed by (Abd Rahman et al., 2017; Song and Morgan, 2019), which included statements such as “We share the inventory level and production plan of food raw material. Food safety trust was measured using a ten-item scale developed by Medeiros et al. (2004) which included statements such as “I am worried that I may get sick if I eat hot dogs right out of the package.” Consumer awareness of organic food was assessed using a two-item scale derived from Asif et al. (2018) which included statements like “Do you know what an organic food is?” We used 5 items to measure the consumer wellbeing from Rudd et al. (2012), which included statements like “In most ways, my life is close to my ideal.”

We employed statistical remedies in order to mitigate the potential effects of *common method bias* (CMB). The findings of the Harman’s one-factor test showed that all of the items could be separated into four constructs based on the criterion of eigenvalues larger than 1. The first component accounted for 44.80% of the variation, and all of them together explained 69.41% of it. The data was therefore suitable for further investigation.

## Results

To assess the concept reliability, we use confirmatory factor analysis (CFA). According to Table 1, every Cronbach’s alpha and composite reliability score is more than 0.7. The validity of the content, convergent, and discriminant measures was also examined. Content validity is supported by the fact that all the items were taken from earlier studies and only minor revisions were made for the research situation. Using factor loadings, composite validity, and average variance extracted (AVE). Convergent validity was evaluated AVEs vary from 0.63 to 0.83 (Table 1), and all of the factor loadings and composite reliability values are close to or higher than the benchmarks, showing robust convergent validity. We then evaluated the discriminant validity of each scale’s primary construct and measurement items. Table 2 demonstrates that for each construct, the square root of the AVE is greater than the correlation coefficient between any two variables, ensuring the discriminant validity (Fornell and Larcker, 1981).

**Table 1:** Confirmatory factor analysis: Validity and reliability.

Latent variables	Standardized loadings	AVE	CR	Alpha
*Food supply chain traceability*		0.56	0.96	0.85
OFSC1	0.715			
OFSC2	0.790			
OFSC3	0.847			
OFSC4	0.839			
OFSC5	0.786			
OFSC6	0.834			
OFSC7	0.723			
OFSC8	0.735			
OFSC9	0.709			
OFSC10	0.750			
OFSC11	0.642			
OFSC12	0.705			
OFSC13	0.656			
OFSC14	0.711			
OFSC15	0.650			
*Food safety trust*		0.50	0.90	0.92
FST1	0.500			
FST2	0.501			
FST3	0.500			
FST4	0.557			
FST5	0.582			
FST6	0.843			
FST7	0.813			
FST8	0.870			
FST9	0.895			
FST10	0.871			
*Consumer awareness*		0.72	0.84	0.91
CA1	0.803			
CA2	0.892			
*Consumer wellbeing*		0.57	0.87	0.82
CW1	0.779			
CW2	0.818			
CW3	0.734			
CW4	0.666			
CW5	0.759			
Full model fit statistics				
*χ*^2^[*df*] = 28.56 [381]; RMSEA = 0.08; NFI = 0.82; TLI = 0.86; CFI = 0.88				

**Table 2:** Discriminant validity test results.

Latent constructs	1	2	3	4
1. OFSC	*0.749*			
2. Food safety trust	0.683	*0.705*		
3. Consumer awareness	0.739	0.405	*0.705*	
4. Consumer wellbeing	0.640	0.640	0.498	0.753

The √ of the average variance extracted was shown on the diagonal.

### Hypotheses testing

Table 3 lists each variable’s mean, standard deviation, and correlation coefficient. There is a substantial positive relationship between organic food supply chain traceability (OFSC) and food safety (*r* = 0.62, *p* < 0.01), organic food supply chain traceability (OFSC) and consumer wellbeing (*r* = 0.59, *p* < 0.01), and food safety trust and consumer wellbeing (*r* = 0.47, *p* < 0.01). Additionally, there is a strong positive association (*r* = 0.35, *p =* 0.01) between consumer wellbeing and consumer awareness of organic food. The study hypothesis is supported by the correlation of key constructs, which offers substantial support for the hypotheses’ validity.

**Table 3:** Means, standard deviations, and correlations for relevant variables.

Variables	Mean	SD	1	2	3	4
1. OFSC	4.65	1.28	1			
2. Food safety	4.81	1.29	0.62**	1		
3. Awareness	4.86	1.55	0.66**	0.36**	1	
4. Wellbeing	4.95	1.16	0.59**	0.47**	0.35**	1

*N* = 221; *p < 0.05; **p < 0.01.

After examining the validity of our research model, all proposed hypotheses were evaluated. The results proposed that OFSC has a significant positive relationship with consumer wellbeing (*B* = 0.44, t = 7.17, *p* < 0.001) with food safety (*B* = 0.62, *t* = 11.65, *p* < 0.001). The results also stated food safety relationship with consumer wellbeing (*B* = 0.15, *t* = 2.44, *p* < 0.001). These findings support our H1, H2, and H3. We followed a two-step method as proposed by Preacher et al. (2007), to test H4 of a mediation role of food safety between OFSC and consumer wellbeing. The results confirmed (Table 4) that the OFSC and consumer wellbeing (*B* = 0.09; *p* < 0.05) are significant *via* the mediation of food safety. Therefore, H4 was also supported.

**Table 4:** Mediation analysis.

		Food safety trust			Consumer wellbeing		
		*B*	*SE*	*p*	*B*	*SE*	*p*
1	OFSC	0.62**	0.05	0.01	0.44***	0.06	0.01
2	Food safety trust	--	--	--	0.15*	0.06	0.05
Indirect effects *via* bootstrap					0.09* (0.01 0.23)		
Indirect effects *via* normal distribution					0.09* (1.99)		

*N* = 221. We report the 95% confidence intervals (CIs) calculated using 5,000 bootstrap samples, with lower and upper limits in brackets. Statistically significant indirect effects are in bold face text. *p < 0.05, **p < 0.01, ***p < 0.001.

**Figure 2:**
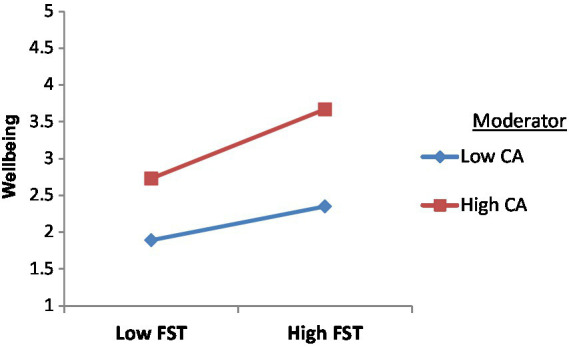
Interaction plot.

According to Hayes and Scharkow, the mediated-moderation effects were tested using the SPSS PROCESS Macro 3.3. The Bootstrapping method was used to analyze the mediated moderating effects (Figure 2). In order to create a generally stable sample combination distribution and accurately assess the sample’s general characteristics, Bootstrap employs the repeated sampling method with the return. There are 5000 Bootstrap samples total, and the confidence interval is set to 95%. The results indicated (Table 5) that the interaction between food safety and consumer awareness had a 0.12 impact on consumer wellbeing, with a confidence range of (0.06, 0.16), which excluded 0. Hence H5 was further verified.

**Table 5:** Mediated-moderation results.

	Food safety				Consumer wellbeing			
	*B*	*SE*	*p*		*B*	*SE*	*p*	
OFSC (X)	0.62	0.05	0.01		0.45	0.07	0.02	
Food safety (M)					0.035	0.13	0.01	
Consumer awareness (W)					0.54	0.13	0.01	
M*W					0.12	0.03	0.01	
	*R*^2^ = 0.38				*R*^2^ = 0.42			
**Moderator**	**Conditional effect of M on Y**				**Conditional effect of X on Y *via* M**			
**Consumer awareness**	***B***	***SE***	**LLCI**	**ULCI**	***B***	***SE***	**LLCI**	**ULCI**
Consumer awareness −1 SD	0.04	0.06	−0.08	0.16	0.03	0.06	−0.09	0.16
Consumer awareness M	0.21	0.06	0.08	0.32	0.13	0.06	0.02	0.26
Consumer awareness +1 SD	0.37	0.08	0.22	0.53	0.23	0.07	0.10	0.39

Table 5 shows that when consumer awareness was stronger, the indirect impact was weaker and insignificant (conditional indirect effect = 0.03, 95 percent CI = [−0.09, 0.16]), but when consumer awareness was lower, the indirect effect was stronger (conditional indirect effect = 0.23, 95%CI = [0.10, 0.39]). Consequently, these findings supported H6.

## Discussion

Our findings provide empirical support in line with Kamble et al. (2020) that incorporating traceability in the FSC can result in consumer satisfaction and that traceability has a good impact on food safety and social sustainability to support the conclusions of Yuan et al. (2020). Additionally, we emphasize those traceability procedures for organic food will enhance consumer wellbeing, which supports the conclusions of Yuan et al. (2020) that traceability can be a useful approach for positively influencing consumers’ perceptions of value. The supply chain stakeholders in our Chinese setting think that adopting a traceability system will raise their customers’ trust and help them improve their customers’ perceptions of the nutritional value, safety, and quality of their organic food items. In recent years, traceability has grown in significance as a research area. This growing research focus has been greatly influenced by the demand for trust and safety, particularly in the organic food traceability, as well as recent assessments of new technologies that are expected to have a considerable impact on traceability practices. As stated in the research question, the purpose of this study was to provide a comprehensive overview of the impact of traceability systems as they pertain to identify the literature and elaborate on the connection between traceability and outcomes. The analytical methodology was adopted from Zhou et al. (2022) and concentrated on organic food products because Zhou et al. (2022) discovered a substantial effect in the food supply chain category in their study. Our findings validate the framework’s effectiveness in a Chinese consumption setting and demonstrate that this analytical methodology is applicable to the category of organic food products. Thus, this study provides a crucial cross-cultural examination of the analytical framework proposed by De Jonge et al. (2008).

This study supports the finding that there is a mediatory mechanism between food supply chain traceability practices and customer wellbeing, which support the findings of Zhou et al. (2022). We choose food safety as a mediator and integrate the theory of dynamic capabilities into the theoretical framework to examine the relationship between organic FSC traceability and consumer wellbeing. This study has shown that organic FSC traceability procedures can increase food safety, which somewhat corroborates the findings of Charan and Panghal (2018) and Aung and Chang (2014), that the traceable assurance can amplify long-term safety concerns, increasing consumer desire due to confidence in food safety. Our findings also suggest that the impact of organic FSC traceability methods on consumer wellbeing is influenced indirectly by improved food safety regulations. Food safety, as a powerful technique of transferring business resources into positive outcomes, explains the mechanisms that link organic FSC traceability practices to consumer wellbeing. Traceability implementation issues can be classified as intra-firm, inter-firm, technical, or external. This study also highlighted that the benefits of implementing traceability may be preceded by a large rise in short-term operating costs as well as inter-firm relationship friction. Financial constraints are a significant barrier to the implementation of traceability systems, and they can manifest themselves in various ways at various stages of traceability implementation, such as the cost of technology (hardware and software); the cost of staff training; and the legal costs associated with information accessibility. This study’s sample companies are from the early benchmarking companies in China that applied organic FSC traceability standards. As a result, their traceable practices can directly influence consumer wellbeing while indirectly promoting a long-term customer base through food safety trust.

The study’s analysis of the function and influence of consumer awareness of organic food on food safety is another important contribution. This empirical finding also addresses the ongoing discussion regarding whether consumer knowledge of traceability and awareness of organic food could lead to such relationships (Hong et al., 2020; Cavite et al., 2021). The empirical research in this paper supports this position by demonstrating that the relationship between food safety trust and consumer wellbeing can be moderated by consumers’ awareness of organic food. Our results reveal that, when individuals are more aware of organic food, food safety has a greater impact on consumer wellbeing. In other words, increased awareness of organic food may result in greater food safety regulations. Firms may take an active role in traceability because they are aware of how customer needs change (Wowak et al., 2016). This study discovered that traceability techniques promote the firm’s food safety concerns, consequently improving consumer wellbeing. Firms should actively promote their safety standards to reap long-term benefits as an intermediary mechanism that partially turns traceable practices into consumer happiness and wellbeing. The present study examines the contingency effect of organic food awareness and improves the interpretation of the process and boundaries of food safety regulations in the setting of China. Furthermore, we contend that there is a connection between food safety and consumer awareness of organic food in the overall effect of organic FSC traceability methods and consumer wellbeing, which may expand the benefits of organic FSC traceability practices.

## Theoretical and managerial implications

As consumer-centric research perspectives with positive outcomes based on traceability procedures are still limited (Cousins et al., 2019; Testa et al., 2019). This research investigates the impact of organic FSC traceability procedures on customer wellbeing and its unique mechanism in the context of Chinese enterprises, with theoretical implications for expanding and deepening research in the field of traceability practices. Based on dynamic capacities theory, this study introduces the mediating influence of food safety and the moderating effect of awareness of organic food between organic FSC traceability practices and consumer wellbeing. This article also offers a fresh theoretical perspective for the study of the organic FSC traceability’s impact mechanism. As a final step, this study also explains the internal mechanism by which consumer awareness may conditionally affect the strength of the indirect relationship between OFST and consumer wellbeing through food safety, indicating a mediated-moderation configuration between the study’s variables. This will help academia gain a deeper and more comprehensive understanding of the process of putting traceability practices into practice with such positive results. Managers can focus more on upstream traceability by emphasizing the source security of raw materials and goods, which is a link that downstream companies and consumers value the most. Consumers still do not trust traceable organic foods at this time, mostly due to the frequent occurrence of food safety issues that substantially affect the reputation of businesses and the absence of an efficient communication channel between markets and traceable organic foods (Bei and Jiabao, 2015). In line with Pattnaik et al. (2021) and Centobelli et al. (2022), given the growing emphasis on Agenda 2030 for Sustainable Development, policymakers and governments could persuade firms to use sustainable supply chain management methods in order to reduce environmental pollution and advance societal welfare.

As a result, food production and sales enterprises can actively expand their visibility, such as inviting citizens to visit food origins or food processing firms, to improve citizens’ opinions of the trustworthiness of these firms and traceability systems. Artificial intelligence, for example, might be utilized to develop a food supply chain traceability system for real-time food tracing, providing a secure and transparent information platform for all supply chain stakeholders. As an intermediary mechanism that turns organic FSC traceable practices into customer wellbeing, businesses should proactively improve food safety standards for the promotion of a healthy food sector development.

## Limitations and future research directions

Even though this study had a sufficient number of respondents in multiple Chinese cities and with various sorts of businesses, its external validity might still be enhanced. Although the present study is usually representative of China, it is not a comprehensive gauge of national organic FSC consumer issues, and China’s food supply chain and consumer habits are distinctive in a number of ways. Future research can utilize cross-regional and cross-cultural samples to confirm and broaden these findings. It would be advantageous to collect data from other nations in order to determine the effect of traceability methods on the sustainable performance of businesses in varied institutional and cultural contexts. Second, this study’s respondents included senior management and technical personnel from the organization. Future research could benefit from responses from other managerial positions of the company, such as selecting the financial directors to investigate the company’s economic performance and the operations or supply chain manager for more precise measurements of social and environmental performance. Future research in this area should test the hypothesis that the higher the level of alignment of an enterprise’s capabilities with traceability practices, the higher the enterprise’s performance (Centobelli et al., 2018). In addition, longitudinal research including follow-up studies would further show the dynamic characteristics if applied to the suggested model.

## Conclusion

As mentioned, earlier studies paid little attention to the assessment of consumer wellbeing in organic food supply chain traceability systems. Based on dynamic capacities theory, this study uses a SEM technique to investigate the impact of OFSC traceability practices on customer wellbeing and examines this link through the mediation of food safety trust and the moderating of consumer awareness of organic food. The findings suggest that OFSC traceability methods have a considerable positive impact on consumer wellbeing, which is also mediated by consumer trust in food safety. Furthermore, consumer awareness of organic food acts as a moderator in the relationship between consumer wellbeing and food safety trust. The greater consumer awareness of organic food, the greater the favorable effects of food safety trust on consumer wellbeing. This study also addresses the research gap by providing an initial empirical examination of the role of consumer awareness conditionally influencing the strength of the indirect relationship between OFST and consumer wellbeing *via* food safety, thereby indicating a mediated-moderation configuration between the study’s variables. Thus, the present study clarifies how organic food traceability and customer trust in food safety can improve wellbeing. It supports organic traceable product marketing tactics as well as the principle of dynamic capabilities. Producers and marketers of organic traceable foods are given guidelines on how to put sensible plans into place to draw customers in and support the long-term growth of the organic food business.

## Data availability statement

The raw data supporting the conclusions of this article will be made available by the authors, without undue reservation.

## Ethics statement

The studies involving human participants were reviewed and approved by Yunnan University, Ethics Approval Committee. The patients/participants provided their written informed consent to participate in this study.

## Author contributions

ZG: conceptualization, methodology, software, validation, formal analysis, writing—original draft preparation, visualization, and supervision. MH: project administration, funding and acquisition, investigation, resources, data curation, and writing—review and editing. All authors have read and agreed to the published version of the manuscript.

## Funding

This research was supported by “National Natural Science Foundation of China: Collaborative Governance on Food Safety in China Under the Perspective of Industry Regulation”, Grant No.: 71964038.

## Conflict of interest

The authors declare that the research was conducted in the absence of any commercial or financial relationships that could be construed as a potential conflict of interest.

## Publisher’s note

All claims expressed in this article are solely those of the authors and do not necessarily represent those of their affiliated organizations, or those of the publisher, the editors and the reviewers. Any product that may be evaluated in this article, or claim that may be made by its manufacturer, is not guaranteed or endorsed by the publisher.
